# *Tlr4*-mutant mice are resistant to acute alcohol-induced sterol-regulatory element binding protein activation and hepatic lipid accumulation

**DOI:** 10.1038/srep33513

**Published:** 2016-09-15

**Authors:** Zhi-Hui Zhang, Xiao-Qian Liu, Cheng Zhang, Wei He, Hua Wang, Yuan-Hua Chen, Xiao-Jing Liu, Xi Chen, De-Xiang Xu

**Affiliations:** 1 Department of Toxicology, Anhui Medical University, Hefei, 230032, China; 2 First Affiliated Hospital, Anhui Medical University, Hefei, 230032, China

## Abstract

Previous studies demonstrated that acute alcohol intoxication caused hepatic lipid accumulation. The present study showed that acute alcohol intoxication caused hepatic lipid accumulation in *Tlr4*-wild-type mice but not in *Tlr4*-mutant mice. Hepatic sterol-regulatory element binding protein (SREBP)-1, a transcription factor regulating fatty acid and triglyceride (TG) synthesis, was activated in alcohol-treated *Tlr4*-wild-type mice but not in *Tlr4*-mutant mice. Hepatic *Fas*, *Acc*, *Scd-1* and *Dgat-2*, the key genes for fatty acid and TG synthesis, were up-regulated in alcohol-treated *Tlr4*-wild-type mice but not in *Tlr4*-mutant mice. Additional experiment showed that hepatic MyD88 was elevated in alcohol-treated *Tlr4*-wild-type mice but not in *Tlr4*-mutant mice. Hepatic NF-κB was activated in alcohol-treated *Tlr4*-wild-type mice but not in *Tlr4*-mutant mice. Moreover, hepatic GSH content was reduced and hepatic MDA level was elevated in alcohol-treated *Tlr4*-wild-type mice but not in *Tlr4*-mutant mice. Hepatic CYP2E1 was elevated in alcohol-treated *Tlr4*-wild-type mice but not in *Tlr4*-mutant mice. Hepatic *p67phox* and *gp91phox*, two NADPH oxidase subunits, were up-regulated in alcohol-treated *Tlr4*-wild-type mice but not in *Tlr4*-mutant mice. Alpha-phenyl-N-t-butylnitrone (PBN), a free radical spin-trapping agent, protected against alcohol-induced hepatic SREBP-1 activation and hepatic lipid accumulation. In conclusion, *Tlr4*-mutant mice are resistant to acute alcohol-induced hepatic SREBP-1 activation and hepatic lipid accumulation.

Alcohol is widely consumed around the world. Chronic alcohol consumption leads to alcoholic liver disease (ALD), characterized by a spectrum of liver injury ranging from simple alcoholic fatty liver to steatohepatitis that leads to hepatic fibrosis and cirrhosis^1^. Alcoholic fatty liver is a relatively benign state of ALD. Hepatic excessive lipid accumulation is the hallmark of alcoholic fatty liver^2^. Excessive fatty acid and triglyceride (TG) synthesis is the main cause of hepatic lipid accumulation in the development of alcoholic fatty liver^3^. Sterol-regulatory element binding protein (SREBP)-1 is an important transcription factor that regulates genes for fatty acid and TG synthesis^4^. Although the immature SREBP-1 is retained in the endoplasmic reticulum, the mature form of SREBP-1 translocates into the nucleus^5^,^6^,^7^,^8^,^9^. Numerous reports support that hepatic SREBP-1 activation plays an important role in the pathogenesis of alcoholic fatty liver^10^,^11^,^12^.

Increasing evidence demonstrates that alcohol consumption impairs the integrity of intestinal mucosa and allows gut-derived bacterial lipopolysaccharide (LPS) to escape into circulation^13^,^14^,^15^. LPS has been proposed as a key player in the pathogenesis of alcoholic fatty liver^16^,^17^. Toll-like receptor 4 (TLR4), expressed mainly on Kupffer cells as well as hepatocytes, acts as a receptor for LPS and mediates production of reactive oxygen species (ROS) and inflammatory cytokines^18^,^19^,^20^. An earlier report study showed that TLR4 was involved in the pathogenesis of chronic alcoholic fatty liver^21^. According to several reports, acute alcohol intoxication caused a sharp elevation of LPS in plasma^22^,^23^. In addition, acute alcohol intoxication induced hepatic lipid accumulation^24^,^25^. Nevertheless, whether TLR4 is involved in acute alcohol-induced hepatic lipid accumulation is not fully understood.

In the present study, we analyzed the effects of acute alcohol intoxication on hepatic lipid accumulation in *Tlr4*-wild-type and *Tlr4*-mutant-type mice. Our results showed that *Tlr4*-mutant mice are resistant to acute alcohol-induced hepatic SREBP-1 activation and hepatic lipid accumulation. We demonstrate for the first time that TLR4-derived ROS is partially involved in acute alcohol-induced hepatic SREBP-1 activation and hepatic lipid accumulation.

## Results

### Acute alcohol intoxication induces hepatic TG accumulation in *Tlr4*-wild-type mice but not in *Tlr4*-mutant mice

**Figure 1:**
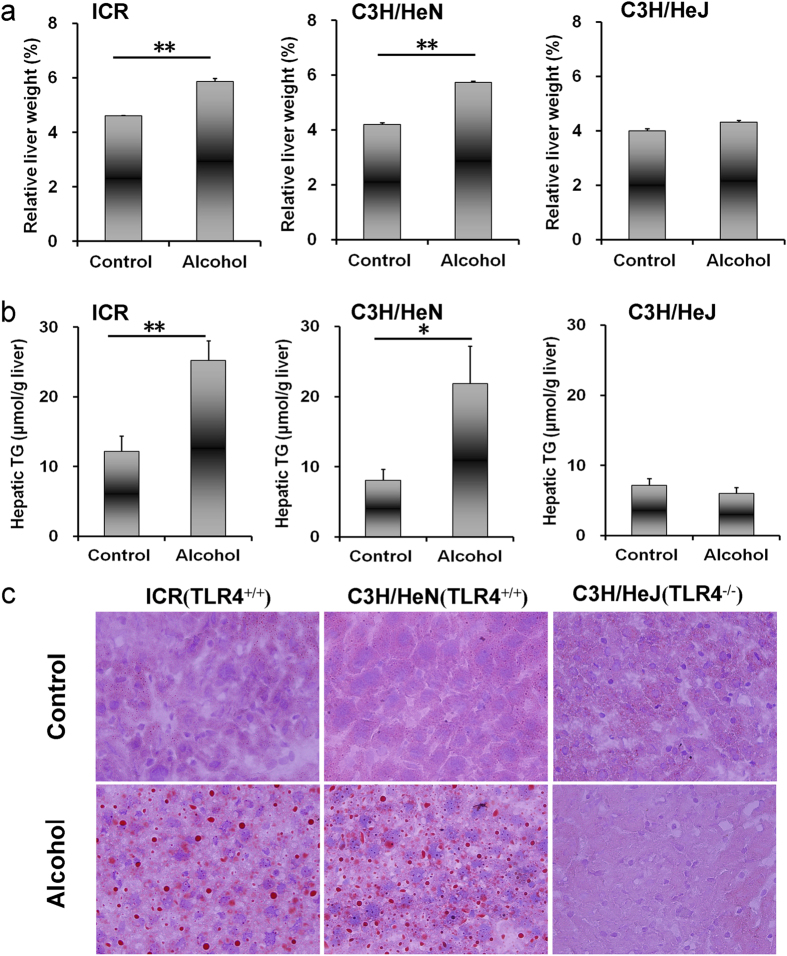
Acute alcohol intoxication induces hepatic TG accumulation in *Tlr4*-wild-type (ICR and C3H/HeN) mice but not in *Tlr4*-mutant-type (C3H/HeJ) mice. All mice except controls were administered with ethanol (4 g/kg) by gavage. Liver tissue was collected 12 h after alcohol. (**a**) Liver weight/body weight ratio. (**b**) Hepatic TG content. (**c**) Liver sections were stained with oil red O. Original magnification, x200. Data were expressed as means ± SEM (N = 6). *P < 0.05, **P < 0.01.

Biochemical parameters were measured 24 h after alcohol exposure. As shown in Table 1, acute alcohol exposure had little effect on serum TG, total cholesterol, total bilirubin, direct bilirubin and total bile acid. Of interest, the absolute liver weight was elevated in alcohol-treated *Tlr4*-wild-type (ICR and C3H/HeN) mice but not in *Tlr4*-mutant-type (C3H/HeJ) mice (Table 1). In addition, liver/body weight ratio was elevated in alcohol-treated *Tlr4*-wild-type (ICR and C3H/HeN) mice but not in *Tlr4*-mutant-type (C3H/HeJ) mice (Fig. 1a). The effects of acute alcohol exposure on hepatic TG and cholesterol were then analyzed. Although acute alcohol exposure had no effect on hepatic cholesterol level (Table 1), it elevated hepatic TG content in *Tlr4*-wild-type (ICR and C3H/HeN) mice but not in *Tlr4*-mutant-type (C3H/HeJ) mice (Fig. 1b). Correspondingly, an obvious hepatic lipid accumulation was observed in alcohol-treated *Tlr4*-wild-type (ICR and C3H/HeN) mice but not in *Tlr4*-mutant-type (C3H/HeJ) mice (Fig. 1c).

### Acute alcohol intoxication activates hepatic SREBP-1 in *Tlr4*-wild-type mice but not in *Tlr4*-mutant mice

**Figure 2:**
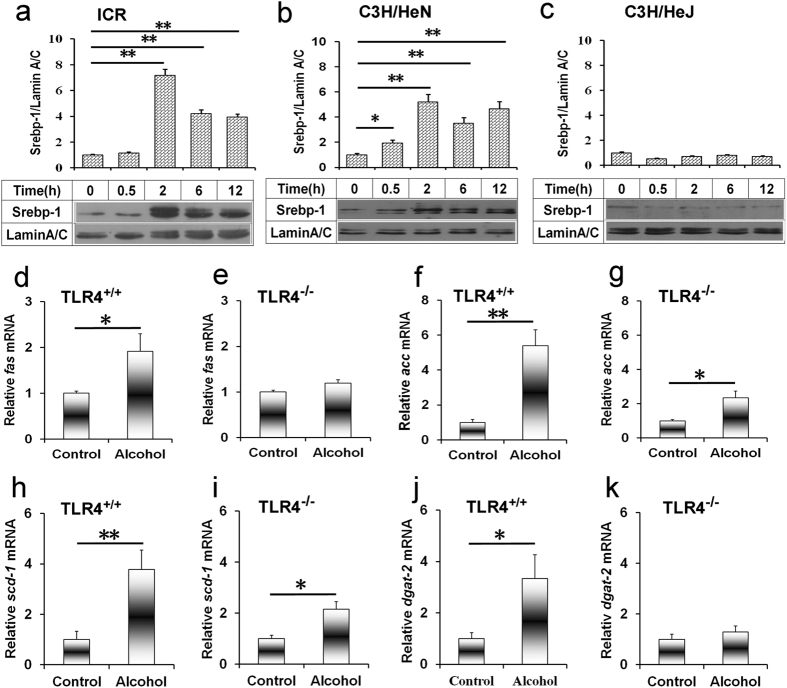
Acute alcohol intoxication activates hepatic SREBP-1 and up-regulates genes for fatty acid and TG synthesis in in *Tlr4*-wild-type mice but not in *Tlr4*-mutant-type mice. All mice except controls were administered with ethanol (4 g/kg) by gavage. (**a–c**) Liver tissue was collected at different time points after alcohol. Hepatic nuclear SREBP-1 was determined using immunoblot. Blots are representatives of six independent experiments. Data were expressed as means ± SEM (N = 6). *P < 0.05, **P < 0.01. (**d–k**) Liver tissue was collected 6 h after alcohol. The expression of (**d,e**) *Fas,* (**f,g**) *Acc,* (**h,i**) *Scd-1,* (**j,k**) *Dgat-2* was determined using real-time RT-PCR. Data were expressed as means ± SEM (N = 6). *P < 0.05, **P < 0.01.

The effects of acute alcohol intoxication on hepatic SREBP-1 activation were analyzed. Hepatic mature SREBP-1 level was elevated in alcohol-treated *Tlr4*-wild-type (ICR and C3H/HeN) mice (Fig. 2a,b) but not in *Tlr4*-mutant-type (C3H/HeJ) mice (Fig. 2c). Hepatic fatty acid synthase (*Fas*), a key enzyme gene for fatty acid synthesis, was up-regulated in alcohol-treated *Tlr4*-wild-type (C3H/HeN) mice (Fig. 2d) but not in *Tlr4*-mutant-type (C3H/HeJ) mice (Fig. 2e). Hepatic acetyl-CoA carboxylase (*Acc*) and stearoyl-CoA desaturase (*Scd*)*-1* was up-regulated by 4~5 fold in alcohol-treated *Tlr4*-wild-type (C3H/HeN) mice (Fig. 2f,h) but only 2 fold in *Tlr4*-mutant-type (C3H/HeJ) mice (Fig. 2g,i). Hepatic diacylglycerol acyltransferase (*Dgat*)*-2*, the key genes for TG synthesis, was elevated in alcohol-treated *Tlr4*-wild-type (C3H/HeN) mice (Fig. 2j) but not in *Tlr4*-mutant-type (C3H/HeJ) mice (Fig. 2k).

### Acute alcohol intoxication induces hepatic Akt activation independent insulin signaling

**Figure 3:**
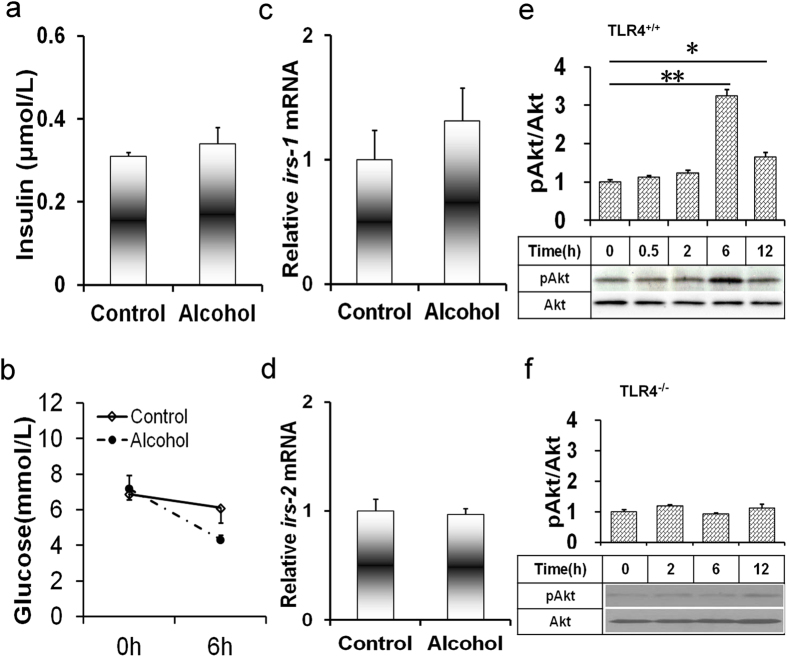
Acute alcohol intoxication induces hepatic PI3K/Akt activation. All mice except controls were administered with ethanol (4 g/kg) by gavage. (**a**) Serum insulin was measured 6 h after alcohol exposure. (**b**) Serum glucose was measured 0 and 6 h after alcohol exposure. (**c,d**) Liver tissues were collected 6 h after alcohol exposure. The expression of hepatic (**c**) *Irs-1* and (**d**) *Irs-2 *mRNAs were determined using real-time RT-PCR. Data were expressed as means ± SEM (N = 6). (**e,f**) Liver tissues were collected at different time points after alcohol exposure. Hepatic Akt and phosphorylated Akt were determined using immunoblot. Blots are representatives of six independent experiments. (**e**) Blots are from *Tlr4*-wild-type mice. (**f**) Blots are from *Tlr4*-mutant-type mice. Data were expressed as means ± SEM (N = 6). **P < 0.01.

As shown in Fig. 3a, acute alcohol exposure had little effect on serum insulin level. Unexpectedly, serum glucose level was significantly reduced 6 h after alcohol exposure (Fig. 3b). To investigate whether acute alcohol intoxication alters hepatic insulin signaling, the expression of hepatic insulin receptor substrate (*Irs*)*-1* and *Irs-2* was measured. As shown in Fig. 3c,d, acute alcohol intoxication had no effect on hepatic *Irs-1* and *Irs-2* mRNA. The effects of acute alcohol intoxication on hepatic Akt phosphorylation were then analyzed. As shown in Fig. 3e,f, hepatic phosphorylated Akt level was elevated in alcohol-treated *Tlr4*-wild-type (C3H/HeN) mice but not in *Tlr4*-mutant-type (C3H/HeJ) mice.

### Acute alcohol intoxication activates hepatic MyD88-dependent TLR4 signaling

**Figure 4:**
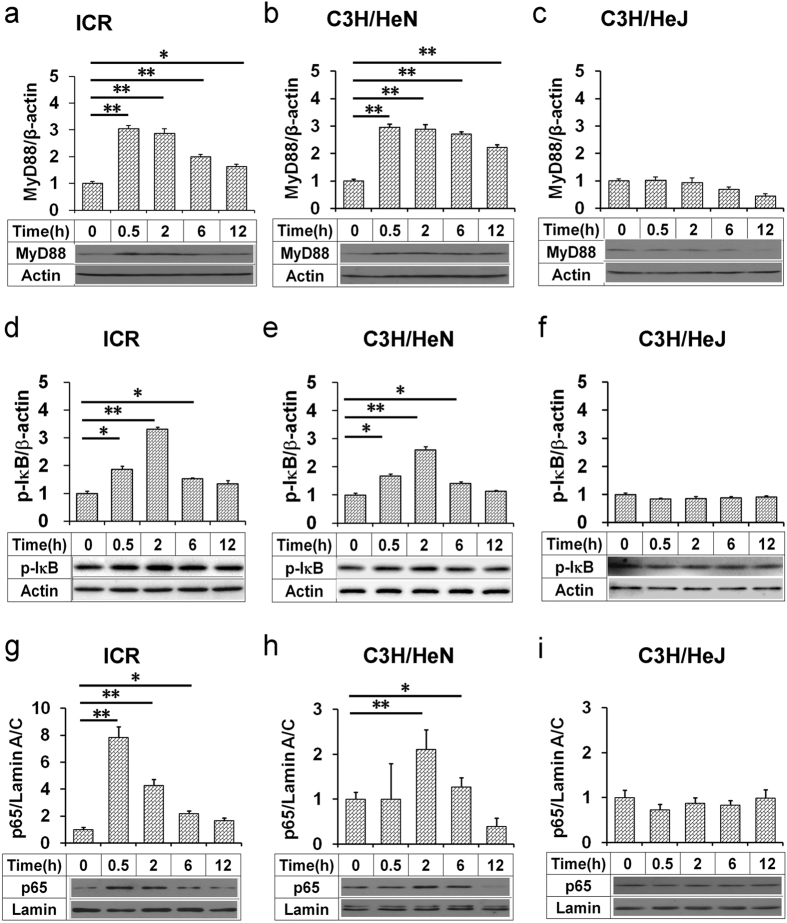
Acute alcohol intoxication activates hepatic TLR4 signaling in *Tlr4*-wild-type (ICR and C3H/HeN) mice but not in *Tlr4*-mutant-type (C3H/HeJ) mice. All mice except controls were administered with ethanol (4 g/kg) by gavage. Liver tissue was collected at different time points after alcohol. Hepatic (**a–c**) MyD88 and (**d–f**) phosphorylated IκBα were determined using immunoblot. (**g–i**) Hepatic nuclear NF-κB p65 was determined using immunoblot. Blots are representatives of six independent experiments. Data were expressed as means ± SEM (N = 6). *P < 0.05, **P < 0.01.

**Figure 5:**
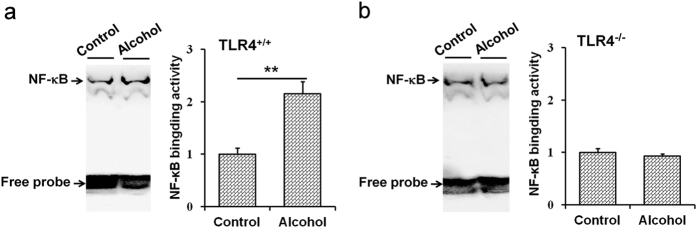
Acute alcohol intoxication elevates hepatic NF-κB binding activity in *Tlr4*-wild-type mice but not in T*lr4*-mutant-type mice. All mice except controls were administered with ethanol (4 g/kg) by gavage. Liver tissue was collected 2 h after ethanol. Hepatic NF-κB binding activity was detected using EMSA. All experiments were repeated for six times. Data were expressed as means ± SEM. (N = 6) **P < 0.01.

The effects of acute alcohol intoxication on hepatic TLR4 signaling were analyzed. As expected, hepatic MyD88 was quickly elevated in alcohol-treated *Tlr4*-wild-type (ICR and C3H/HeN) mice (Fig. 4a,b) but not in *Tlr4*-mutant-type (C3H/HeJ) mice (Fig. 4c). The level of hepatic phosphorylated IκBα was increased in alcohol-treated *Tlr4*-wild-type (ICR and C3H/HeN) mice (Fig. 4d,e) but not in *Tlr4*-mutant-type (C3H/HeJ) mice (Fig. 4f). The level of nuclear NF-κB p65 was elevated in alcohol-treated *Tlr4*-wild-type (ICR and C3H/HeN) mice (Fig. 4g,h) but not in *Tlr4*-mutant-type (C3H/HeJ) mice (Fig. 4i). The effects of acute alcohol intoxication on hepatic NF-κB binding activity was then analyzed. As shown in Fig. 5, hepatic NF-κB binding activity was elevated in alcohol-treated *Tlr4*-wild-type (C3H/HeN) mice (Fig. 5a) but not in *Tlr4*-mutant-type (C3H/HeJ) mice (Fig. 5b). To investigate whether acute alcohol exposure activates hepatic MyD88-independent TLR4 signaling, hepatic interferon regulatory factor (*Irf*)*-3* and *Irf-7* mRNAs were measured. As expected, acute alcohol exposure had little effect on hepatic *Irf-3* and *Irf-7* mRNA (data not shown).

### Acute alcohol intoxication induces hepatic oxidative stress in *Tlr4*-wild-type but not *Tlr4*-mutant-type mice

**Figure 6:**
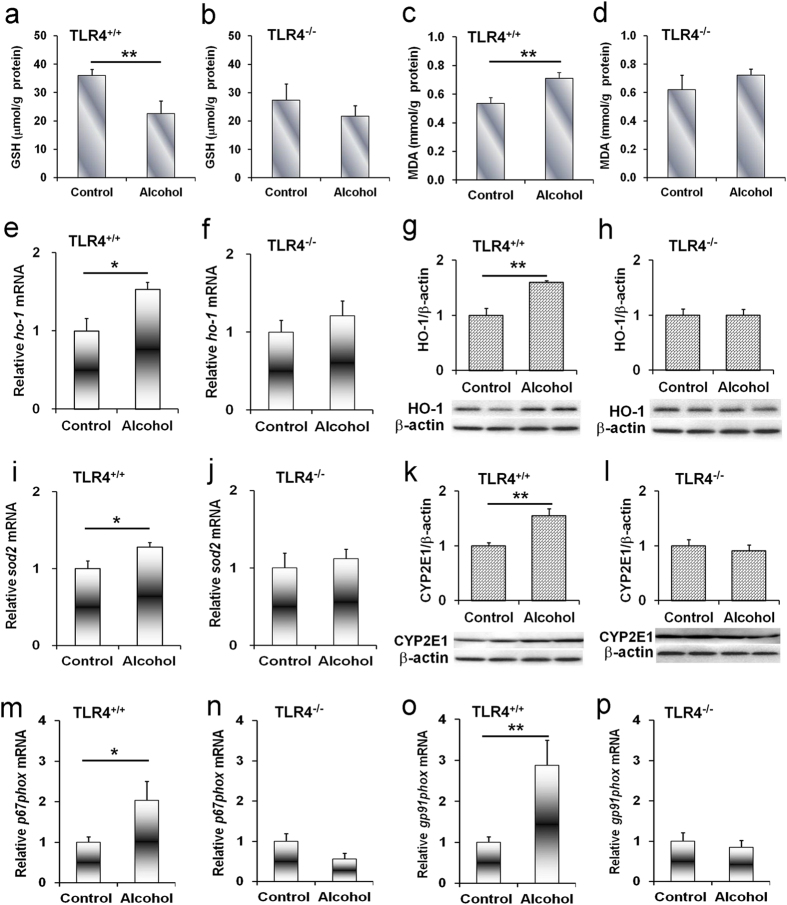
Acute alcohol exposure induces hepatic oxidative stress in *Tlr4*-wild-type mice but not in *Tlr4*-mutant-type mice. All mice except controls were administered with ethanol (4 g/kg) by gavage. (**a–l**) Liver tissue was collected 6 h after alcohol. (**a,b**) Hepatic GSH content. (**c,d**) Hepatic MDA content. The expression of hepatic (**e,f**) *Ho-1* and (**i,j**) *Sod2* mRNA was determined using real-time RT-PCR. Data were expressed as means ± SEM (N = 6). Hepatic (**g,h**) HO-1 and (**k,l**) CYP2E1 proteins were determined using immunoblot. Blots are representatives of three independent experiments. (**m–p**) Liver tissue was collected at 2 h after alcohol. The expression of hepatic (**m,n**) *p67phox* and (**o,p**) *gp91phox* mRNA was determined using real-time RT-PCR. All data were expressed as means ± SEM (N = 6). *P < 0.05, **P < 0.01.

The effects of acute alcohol intoxication on hepatic oxidative stress are presented in Fig. 6. As expected, hepatic GSH content was significantly reduced in alcohol-treated *Tlr4*-wild-type (C3H/HeN) mice (Fig. 6a) but not in *Tlr4*-mutant-type (C3H/HeJ) mice (Fig. 6b). In contrast, hepatic MDA level was elevated in alcohol-treated *Tlr4*-wild-type (C3H/HeN) mice (Fig. 6c) but not in *Tlr4*-mutant-type (C3H/HeJ) mice (Fig. 6d). The effects of acute alcohol intoxication on hepatic *Ho-1* and *Sod-2* mRNAs were then analyzed. As expected, hepatic *Ho-1* and *Sod-2* mRNAs were up-regulated in alcohol-treated *Tlr4*-wild-type (C3H/HeN) mice (Fig. 6e,i) but not in *Tlr4*-mutant-type (C3H/HeJ) mice (Fig. 6f,j). The level of hepatic HO-1 protein was elevated in alcohol-treated *Tlr4*-wild-type (C3H/HeN) mice (Fig. 6g) but not in *Tlr4*-mutant-type (C3H/HeJ) mice (Fig. 6h).

### Acute alcohol intoxication induces hepatic CYP2E1 in *Tlr4*-wild-type but not *Tlr4*-mutant-type mice

The effects of acute alcohol intoxication on hepatic CYP2E1 were analyzed. As expected, hepatic CYP2E1 was elevated in alcohol-treated *Tlr4*-wild-type (C3H/HeN) mice (Fig. 6k) but not in *Tlr4*-mutant-type (C3H/HeJ) mice (Fig. 6l).

### Acute alcohol intoxication induces hepatic NADPH oxidase subunits in *Tlr4*-wild-type but not *Tlr4*-mutant-type mice

The effects of acute alcohol intoxication on hepatic *p67phox*, *gp91phox*, *p22phox* and *p47phox*, four NADPH oxidase subunits, were analyzed. As expected, hepatic *p67phox* mRNA was up-regulated in alcohol-treated *Tlr4*-wild-type (C3H/HeN) mice (Fig. 6m) but not in *Tlr4*-mutant-type (C3H/HeJ) mice (Fig. 6n). Hepatic *gp91phox* mRNA was elevated in alcohol-treated *Tlr4*-wild-type (C3H/HeN) mice (Fig. 6o) but not in *Tlr4*-mutant-type (C3H/HeJ) mice (Fig. 6p). Acute alcohol exposure did not affect hepatic *p22phox* and *p47phox* expression (Supplementary Fig. S1). Finally, the effects of acute alcohol intoxication on hepatic *nox4* expression were analyzed. As expected, acute alcohol exposure had no effect on hepatic *nox4* expression (data not shown).

### PBN protects against acute alcohol-induced hepatic lipid accumulation

**Figure 7:**
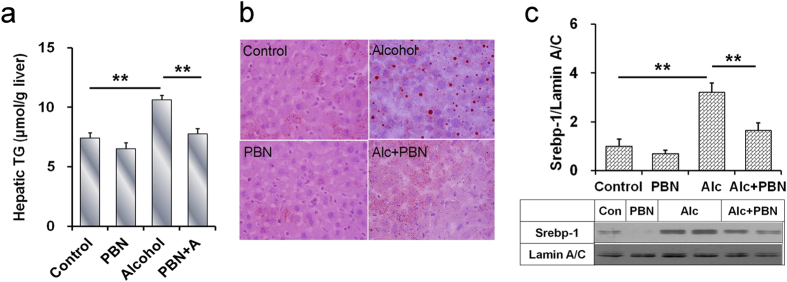
PBN protects against acute alcohol-induced hepatic SREBP-1 activation and hepatic TG accumulation. PBN and alcohol were administered as Materials and Methods. Liver tissue was collected 6 h after alcohol. (**a**) Hepatic TG content. (**b**) Liver sections were stained with oil red O. Original magnification, x200. (**c**) Hepatic nuclear SREBP-1 was determined using immunoblot. Data were expressed as means ± SEM (N = 6). **P < 0.01.

The effects of PBN, a free radical spin-trapping agent, on alcohol-induced hepatic lipid accumulation were analyzed. As expected, PBN alone did not affect hepatic TG content (Fig. 7a). In addition, PBN alone did not induce hepatic lipid accumulation (Fig. 7b). Of interest, alcohol-induced elevation of hepatic TG content was attenuated in mice pretreated with PBN (Fig. 7a). Alcohol-evoked hepatic TG accumulation was alleviated by PBN pretreatment (Fig. 7b). Further analysis showed that alcohol-induced hepatic SREBP-1 activation was attenuated by PBN pretreatment (Fig. 7c).

## Discussion

The present study showed that hepatic TG content was elevated in alcohol-treated *Tlr4*-wild-type mice but not in *Tlr4*-mutant mice. An obvious hepatic lipid accumulation, as determined by Oil red O staining, was observed in alcohol-treated *Tlr4*-wild-type mice but not in *Tlr4*-mutant mice. Increasing evidence has demonstrated that de novo fatty acid and TG synthesis plays an important role in hepatic lipid accumulation^3^. SREBP-1c is an important transcription factor that regulates genes for hepatic fatty acid and TG synthesis^26^. The present study showed that hepatic SREBP-1 was activated in alcohol-treated *Tlr4*-wild-type mice but not in *Tlr4*-mutant mice. Several key genes for hepatic fatty acid and TG synthesis were up-regulated in alcohol-treated *Tlr4*-wild-type mice but not in *Tlr4*-mutant mice. These results suggest that *Tlr4*-mutant mice are resistant to acute alcohol-induced hepatic SREBP-1 activation and hepatic lipid accumulation.

TLR4 activates two signaling pathways through different adapter molecules: MyD88 and TRIF^20^. The present study showed that hepatic NF-κB binding activity was elevated in alcohol-treated *Tlr4*-wild-type mice but not in *Tlr4*-mutant mice. Hepatic pIκBα was elevated in alcohol-treated *Tlr4*-wild-type mice but not in *Tlr4*-mutant mice. Nuclear translocation of hepatic NF-κB p65 subunit was observed in alcohol-treated *Tlr4*-wild-type mice but not in *Tlr4*-mutant mice. These results suggest that hepatic NF-κB, a downstream molecule of TLR4 signaling, was activated in alcohol-treated *Tlr4*-wild-type mice but not in *Tlr4*-mutant mice. Of interest, acute alcohol exposure had no effect on hepatic *Irf-3* and *Irf-7*, two downstream genes of TRIF signaling. Taken together, these results indicate that acute alcohol exposure activates hepatic MyD88-dependent but TRIF-independent TLR4 signaling.

Several studies indicate that excess ROS promote hepatic SREBP-1c activation and hepatic lipid accumulation^27^,^28^. Hepatic NADPH oxidase is the major source of ROS in alcohol-intoxicated mice^29^. According to an earlier report, hepatic *p22phox*, *gp91phox*, *p47phox*, and *p67phox*, four NADPH oxidase subunits, were up-regulated in chronic alcohol-exposed mice^21^,^30^. The present study investigated the effects of acute alcohol intoxication on hepatic NADPH oxidase subunits. Although acute alcohol intoxication had little effect on hepatic *p22phox* and *p47phox* expression, hepatic *p67phox* and *gp91phox*, two NADPH oxidase subunits, were up-regulated in alcohol-intoxicated *Tlr4*-wild-type mice but not in *Tlr4*-mutant-type mice. Thus, we hypothesize that NADPH oxidase-derived ROS may be involved in acute alcohol-intoxicated hepatic SREBP-1c activation and hepatic lipid accumulation. To demonstrate this hypothesis, we investigated the effect of PBN, a free radical spin-trapping agent, on alcohol-induced hepatic SREBP-1c activation and hepatic lipid accumulation. As expected, acute alcohol-evoked hepatic SREBP-1 activation was attenuated in PBN-pretreated mice. Acute alcohol-induced elevation of hepatic TG content was alleviated by PBN pretreatment. In addition, acute alcohol-induced hepatic lipid accumulation was blocked by PBN pretreatment. These results suggest that ROS, probably sourced from NADPH oxidase, a downstream molecule of TLR4 signaling, contribute to acute alcohol-evoked hepatic SREBP-1 activation and hepatic lipid accumulation.

CYP2E1 is another major source of hepatic ROS production^31^,^32^,^33^. Several studies demonstrated that hepatic CYP2E1 was up-regulated in chronic alcohol-exposed mice^34^,^35^. The present study found that hepatic CYP2E1 was elevated in alcohol-treated *Tlr4*-wild-type mice. These results are in agreement with an earlier report^36^, in which CYP2E1 catalytic activity was elevated by 2-fold in alcohol-intoxicated mice. Of interest, the present study showed that hepatic CYP2E1 level was not elevated in alcohol-treated *Tlr4*-mutant-type mice. Therefore, the present study does not exclude the role of CYP2E1-sourced ROS in acute alcohol-evoked hepatic SREBP-1 activation and hepatic lipid accumulation.

The mechanism through which ROS mediates hepatic SREBP-1 activation and hepatic lipid accumulation remains obscure. According to an earlier report, excess ROS promoted hepatic phosphatase and tensin homolog deleted on chromosome ten (PTEN) oxidation and subsequent phosphoinositide 3-kinase (PI3K) activation^37^. Another study demonstrated that hepatic SREBP-1 maturation was tightly associated with PI3K activation and subsequent Akt phosphorylation^38^. Indeed, the present study showed that hepatic pAkt level was elevated in alcohol-treated *Tlr4*-wild-type mice but not in *Tlr4*-mutant-type mice. It is well known that insulin not only elevates hepatic SREBP-1 expression but also promotes hepatic SREBP-1 maturation^9^,^39^,^40^,^41^,^42^. Unexpectedly, the present study showed that acute alcohol intoxication did not increase serum insulin level. Moreover, acute alcohol intoxication did not up-regulate hepatic *Irs-1* and *Irs-2* expression, indicating that acute alcohol-evoked hepatic Akt phosphorylation and SREBP-1 activation are independent of insulin signaling. These results suggest that ROS-mediated hepatic Akt phosphorylation may be associated with acute alcohol-evoked hepatic SREBP-1 activation and hepatic lipid accumulation.

In summary, the present study investigated the role of TLR4 on acute alcohol-induced hepatic lipid accumulation. Our results showed that acute alcohol intoxication caused hepatic lipid accumulation in *Tlr4*-wild-type mice but not in *Tlr4*-mutant-type mice. Moreover, acute alcohol intoxication induced hepatic SREBP-1c activation in *Tlr4*-wild-type mice but not in *Tlr4*-mutant-type mice. The present study demonstrates for the first time that TLR4-mediated excess ROS generation, probably sourced from NADPH oxidase, contribute, at least partially, to acute alcohol-evoked hepatic SREBP-1 activation and hepatic lipid accumulation.

## Materials and Methods

### Reagents

Alpha-phenyl-N-t-butylnitrone (PBN) and ethanol were from Sigma Chemical Co. (St. Louis, MO). Antibodies against Akt, pAkt and CYP2E1 were from Cell Signaling Technology (Beverley, MA). Antibodies against HO-1, SREBP-1, NF-κB p65, p-IκB, MyD88, β-actin and Lamin A/C were from Santa Cruz Biotechnologies (Santa Cruz, CA). TRI reagent was from Molecular Research Center, Inc (Cincinnati, Ohio). RNase-free DNase was from Promega Corporation (Madison, WI). Chemiluminescence detection kit was from Pierce Biotechnology (Rockford, IL). Oil Red O was from Sigma Chemical Co. (St. Louis, MO). All other reagents were purchased from Sigma Chemical Co. (St. Louis, MO) if not otherwise stated.

### Animals and treatments

Male CD-1 (*Tlr4*-wild-type, 6~8 week-old; 24~28 g) and C3H/HeN (*Tlr4*-wild-type, 6~8 week-old; 20~22 g) mice were purchased from Beijing Vital River whose foundation colonies were all introduced from Charles River Laboratories, Inc. C3H/HeJ (*Tlr4*-mutant-type) mice were purchased from Nanjing Biomedical Research Institute of Nanjing University (Nanjing, China). The animals were maintained on a 12-h light/dark cycle in a controlled temperature (20–25 °C) and humidity (50 ± 5%) environment for a period of 1 week before use. All mice were fed with regular diet. The food consisted of standard rodent chow (50.76% carbohydrate, 15.44% fat, 33.8% protein calories), which were purchased from Jiangsu Cooperative Medical Biological Engineering Company Limited (Nanjing, China). The present study consisted of two independent experiments. Experiment 1, to investigate the effects of acute alcohol exposure on hepatic lipid accumulation, thirty-six mice each strain (ICR, C3H/HeN and C3H/HeJ) were divided into six groups. For five alcohol-treated groups, mice were administered with a single dose of ethanol (4 g/kg) by gavage. Six mice each strain were sacrificed at each time point (0, 0.5, 2, 6 and 12 h after alcohol). Experiment 2, to investigate the effects of PBN on alcohol-induced hepatic SREBP-1c activation and hepatic lipid accumulation, twenty-four ICR mice were divided into four groups. In alcohol-treated group, mice were administered with a single dose of ethanol (4 g/kg) by gavage. In PBN and PBN + alcohol groups, mice were injected with two doses of PBN (100 mg/kg), one 30 min before alcohol and the other 4 h after alcohol. The alcohol dose used in the present study referred to others with minor modification^43^,^44^. Mice were sacrificed 12 h after alcohol exposure. For all mice, blood serum was collected for measurement of biochemical parameters. Liver was collected and frozen immediately in liquid nitrogen for reverse transcription polymerse chain reaction (RT-PCR), immunoblot and hepatic TG measurement. Some liver tissues were frozen-fixed in optimum cutting temperature compound mounting media for Oil red O staining. All procedures on animals followed the guidelines for humane treatment set by the Association of Laboratory Animal Sciences and the Center for Laboratory Animal Sciences at Anhui Medical University.

### Biochemical parameters

The levels of serum TG, total cholesterol, serum glucose, alanine aminotransferase (ALT), aspartate aminotransferase (AST), total bilirubin, direct bilirubin and total bile acid were measured using a commercially available kit.

### Hepatic TG measurement

Hepatic TG was extracted using method developed by Bligh with minor modification^45^. Briefly, liver samples were homogenized in ice-cold 2 × PBS. TG was extracted with methanol/chloroform (1:2), dried, and resuspended in 5% fat-free bovine serum albumin. Hepatic TG was measured using the TG reagent kit (Zhejiang Dongou Diagnostics Co., LTD) according to manufacturer’s protocol. Hepatic TG content was expressed as μmol/g liver.

### Isolation of total RNA and real-time RT-PCR

Total RNA was extracted from liver tissues using TRI reagent (Molecular Research Center). RNase-free DNase-treated total RNA (1.0 μg) was reverse-transcribed with AMV (Promega). Real-time RT-PCR was performed with a LightCycler^®^ 480 SYBR Green I kit (Roche Diagnostics GmbH, Mannheim, Germany) using gene-specific primers as listed in Supplementary Table S1. According to a recent report, housekeeping gene variability was observed in the liver of alcoholic patients, in which β-actin and GAPDH tended to decrease with steatosis and to increase with alcoholic hepatitis^46^. In liver of alcoholic patients, the most constantly expressed housekeeping gene is *18S*. Our preliminary experiments showed that hepatic *β-actin* and *Gapdh* mRNAs were up-regulated in alcohol-exposed mice. By contrary, hepatic *18S* had the lowest coefficient of dispersion (Supplementary Table S2). Thus, 18S is an appropriate reference gene for normalization of real-time RT-PCR. The amplification reactions were carried out on a LightCycler^®^ 480 Instrument (Roche Diagnostics GmbH) with an initial hold step (95 °C for 5 minutes) and 50 cycles of a three-step PCR (95 °C for 15 seconds, 60 °C for 15 seconds, 72 °C for 30 seconds). The comparative CT-method was used to determine the amount of target, normalized to an endogenous reference (18S) and relative to a calibrator using the LightCycler 480 software (Roche, version 1.5.0)^47^. All RT-PCR experiments were performed in triplicate.

### Immunoblots

Hepatic lysate was prepared by homogenizing 50 mg liver tissue in 300 μl lysis buffer (50 mM Tris-HCl, pH 7.4, 150 mM NaCl, 1 mM EDTA, 1% Triton X-100, 1% sodium deoxycholate, 0.1% sodium dodecylsylphate, 1 mM phenylmethylsulfonyl fluoride) supplemented with a cocktail of protease inhibitors (Roche). For nuclear protein extraction, hepatic lysate was suspended in hypotonic buffer and then kept on ice for 15 min. The suspension was then mixed with detergent and centrifuged for 30 s at 14,000 × g. The nuclear pellet obtained was resuspended in complete lysis buffer in the presence of the protease inhibitor cocktail, incubated on ice for 30 min, and centrifuged for 10 min at 14,000 × g. Protein concentrations were determined with BCA protein assay (Pierce, Rockford, IL, USA) according to manufacturer’s instructions. For immunoblots, same amount of protein (40~80 μg) was separated electrophoretically by SDS-PAGE and transferred to a polyvinylidene fluoride membrane. The membranes were incubated for 2 h with the following antibodies: p-Akt (1:2000), Akt (1:3000), MyD88 (1:1000), p-IκB (1:1000), NF-κB p65 (1:1000), SREBP-1 (1:1000), HO-1(1:1000) and CYP2E1 (1:2000). For total protein, β-actin (1:3000) was used as a loading control. For nuclear protein, lamin A/C (1:2000) was used as a loading control. After washes in DPBS containing 0.05% Tween-20 four times for 10 min each, the membranes were incubated with goat anti–rabbit IgG or goat anti–mouse antibody for 2 h. The membranes were then washed for four times in DPBS containing 0.05% Tween-20 for 10 min each, followed by signal development using an ECL detection kit.

### Oil red O staining

To determine hepatic lipid accumulation, frozen sections of liver (10 μm) were stained with Oil Red O for 10 min, washed, and counterstained with hematoxylin for 45 seconds. Representative photomicrographs were captured at 400x magnification using a system incorporated in the microscope.

### Enzyme-linked immunosorbent assay

Commercial enzyme-linked immunosorbent assay (ELISA) kit (Millipore) was used to determine serum insulin level according to manufacturer’s protocol.

### Electrophoretic mobility shift assay (EMSA)

Nuclear extracts were prepared form liver tissue by the protocol established by Derckere and Gannon^48^. Ten microgram nuclear extracts were incubated at room temperature for 20 min with a biotin-labeled double-strand DNA probe (Sangon Biological Technology, Shanghai, China) containing an NF-kB binding site (5′-AGT TGA GGG GAC TTT CCC AGG C-3′ and 5′-GCC TGG GAA AGT CCC CTC AAC T-3′) in binding buffer (2.5% glycerol, 5 mM MgCl2, 0.05% NP-40, 0.5 mM EDTA [pH 8.0], 0.5 mM DTT, 50 mM NaCl, 10 mM Tris [pH 7.5], and 50 ng/μl poly[dI-dC]). EMSA was performed using a LightShift Chemiluminescence EMSA kit (Pierce Biotechnology, Inc, Rockfor, IL).

### Glutathione assay

The glutathione (GSH) in liver tissue homogenate was determined by the method of Griffith^49^. Briefly, 0.3 mL supernatant of 20% liver homogenate was added in 0.1 mL of 20% trichloroacetic acid, and samples were centrifuged at 4000 rpm for 15 min. Then, 0.1 mL supernatant was combined with 4.4 mL 0.3 M Na2HPO4 and 0.5 mL of 0.04% dithio-bis-nitrobenzoic acid (DTNB), and the absorbance of the solution was read at 412 nm. The content of GSH was expressed as micromole per gram protein.

### Lipid peroxidation assay

Hepatic lipid peroxidation was evaluated by measuring malondialdehyde (MDA) as described previously^50^. To prepared 10% liver homogenate, 0.1 gram liver tissue was homogenized in 1 mL saline and samples were centrifuged at 4000 rpm for 15 min. After that, 0.1 mL supernatant was added into the reaction mixture containing 0.1 mL sodium dodecylsulfate (8.1%), 1.5 mL acetic acid (20%, v/v), 1.5 mL thiobarbituric acid (0.9%). All the tubes were placed in a boiling water bath for 1 hour. After cooling on ice, the reaction mixture centrifuged at 10,000 × g for 10 min. The amount of MDA per sample was assessed by determining the absorbance of the supernatant at 532 nm using tetraethoxypropane as standard. MDA contant was expressed as micromole per gram protein.

### Statistical analysis

All data were expressed as means ± SEM. SPSS 13.0 statistical software was used for statistical analysis. All statistical tests were two-sided using an alpha level of 0.05. ANOVA and the Student-Newmann-Keuls post hoc test were used to determine differences among different groups. Student *t* test was used to determine differences between two groups.

### Ethics statement

This study was approved by the Association of Laboratory Animal Sciences and the Center for Laboratory Animal Sciences at Anhui Medical University (Permit Number: 12-0010). All procedures on animals followed the guidelines for humane treatment set by the Association of Laboratory Animal Sciences and the Center for Laboratory Animal Sciences at Anhui Medical University.

## Additional Information

**How to cite this article**: Zhang, Z.-H. *et al.* *Tlr4*-mutant mice are resistant to acute alcohol-induced sterol-regulatory element binding protein activation and hepatic lipid accumulation. *Sci. Rep.* **6**, 33513; doi: 10.1038/srep33513 (2016).

## Supplementary Material

Supplementary Information

## Figures and Tables

**Table 1:** Physiologic and biochemical parameters.

	ICR		C3H/HeN		C3H/HeJ	
	Control	Alcohol	Control	Alcohol	Control	Alcohol
Liver weight (g)	1.24 ± 0.02	1.58 ± 0.06**	0.93 ± 0.03	1.27 ± 0.03**	0.88 ± 0.02	0.97 ± 0.03
Hepatic cholesterol (μmol/g liver)	5.75 ± 0.33	5.80 ± 0.23	6.05 ± 0.40	6.47 ± 0.41	6.30 ± 1.24	6.20 ± 0.32
Triglyceride (mmol/L)	0.58 ± 0.16	1.03 ± 0.26	0.60 ± 0.04	0.66 ± 0.03	0.53 ± 0.08	0.54 ± 0.06
Total cholesterol (mmol/L)	3.53 ± 0.22	3.26 ± 0.17	3.78 ± 0.13	3.08 ± 0.11	2.87 ± 0.30	1.64 ± 0.31
Serum glucose (mmol/L)	4.16 ± 0.60	5.37 ± 0.84	4.09 ± 0.52	5.23 ± 0.27	4.72 ± 0.93	5.28 ± 1.02
Alanine aminotransferase (ALT, IU/L)	39.00 ± 9.26	77.75 ± 7.38*	40.50 ± 11.67	47.00 ± 12.29	42.25 ± 6.14	57.00 ± 3.70
Aspartate aminotransferase (AST, IU/L)	151.00 ± 15.90	188.75 ± 16.31	119.00 ± 27.58	127.00 ± 11.80	157.75 ± 22.33	215.50 ± 6.72
Total bilirubin (mmol/L)	1.04 ± 0.32	0.80 ± 0.14	1.52 ± 0.05	1.01 ± 0.09	0.60 ± 0.11	0.84 ± 0.20
Direct bilirubin (mmol/L)	0.45 ± 0.15	0.46 ± 0.06	0.61 ± 0.14	0.44 ± 0.06	0.58 ± 0.05	0.67 ± 0.03
Total bile acid (mmol/L)	1.00 ± 0.14	1.08 ± 0.20	1.75 ± 0.32	1.33 ± 0.14	1.53 ± 0.37	1.70 ± 0.13

Data are means ± S.E.M. *P < 0.05, **P < 0.01 vs control group.
