# Supplementary material for: Tlr4-mutant mice are resistant to acute alcohol-induced sterol-regulatory element binding protein activation and hepatic lipid accumulation
Source: Sci Rep. 2016 Sep 15;6:33513. doi: 10.1038/srep33513 (PMC5024165; doi:10.1038/srep33513)
Supplement: Supplementary Information [file srep33513-s1.pdf]

Supplementary information

Tlr4-mutant mice are resistant to acute alcohol-induced sterol-regulatory element binding protein activation and hepatic lipid accumulation

Zhi-Hui Zhang[1, †], Xiao-Qian Liu[1, †], Cheng Zhang[1], Wei He[2], Hua Wang[1], Yuan-Hua Chen[1], Xiao-Jing Liu[2], Xi Chen[2,*], De-Xiang Xu[1,*]

[1]Department of Toxicology, Anhui Medical University, Hefei, 230032, China
[2] First Affiliated Hospital, Anhui Medical University, Hefei, 230032, China

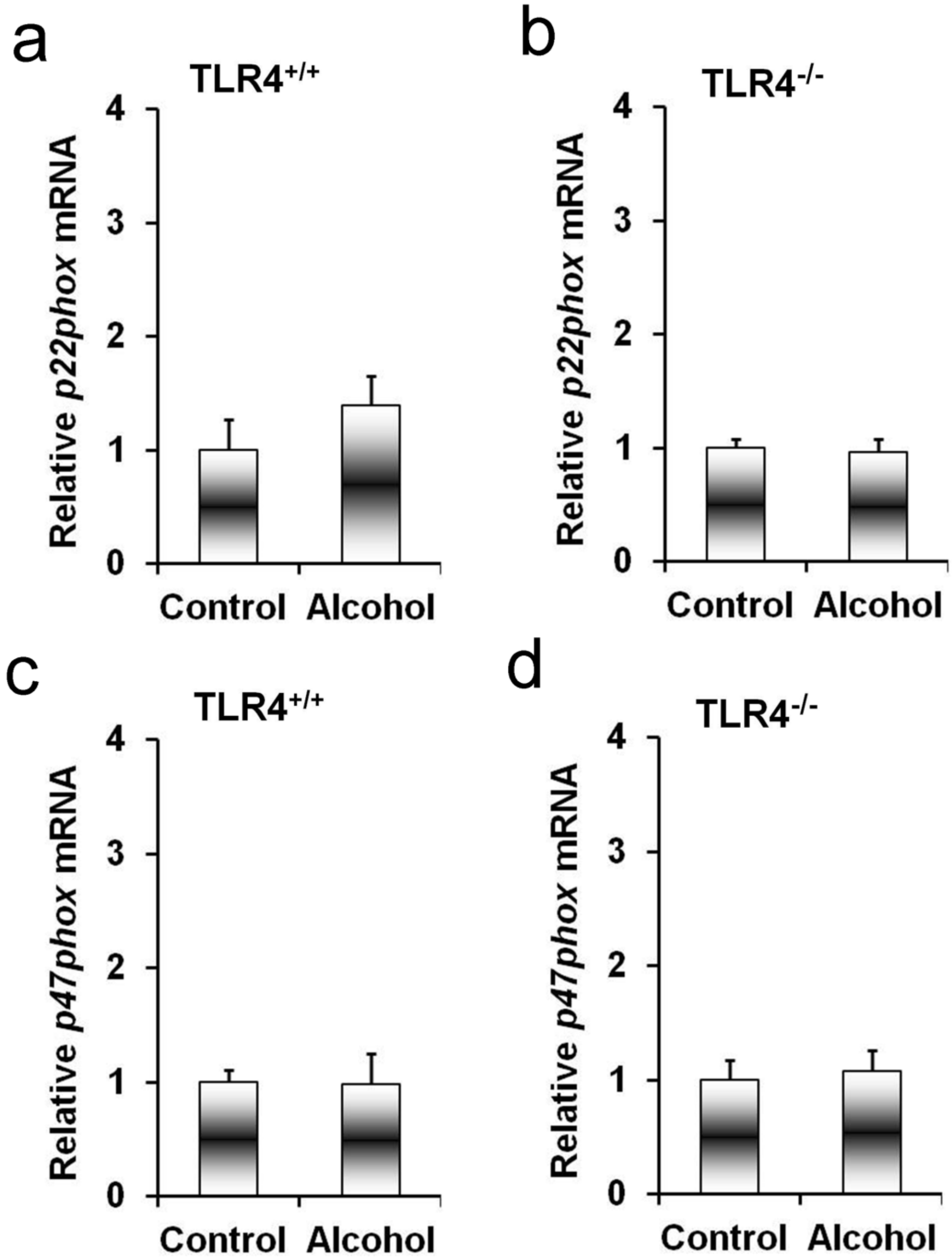

Supplementary Figure S1. Effects of acute alcohol exposure on hepatic NADPH oxidase subunits. All mice except controls were administered with ethanol (4 g/kg) by gavage. Liver tissue was collected at 2 h after alcohol. The expression of hepatic (**a,b**) *p22phox* and (**c,d**) *p47phox* mRNA was determined using real-time RT-PCR. Data were expressed as means ± SEM (N=6).

Supplementary Table S1. Oligonucleotide sequence of primers for real-time RT-PCR

Genes	Forward (5'-3')	Reverse (5'-3')
18S	GTAACCCGTTGAACCCCATT	CCATCCAATCGGTAGTAGCG
fas	CGCTCGGCTCGATGGCTCAG	CCAGCACCACGGCATGCTCA
acc	CCGTTGGCCAAAACTCTGGAGCTAA	GAGCTGACGGAGGCTGGTGACA
scd-1	CGGTCATCCCATCGCCTGCTCT	GTAGGCGAGTGGCGGAACTGC
dgat-2	GGATGCCTGTGCTTCGCGAGT	GCTGCACCTCCCACCACGATG
irs-1	AAAAGCCACTTTCCCCGCCCG	CCACCAGCCGCCCAAATACCA
irs-2	GCGGAGTGTGTGGGGGTCCA	GGGGCTGGTAGCGCTTCACT
ho-1	CGTCACTTCGTCAGAGGCCTGC	TCTGGGGTTTCCCTCGGGGTG
sod-2	AGCGAACGGCCGTGTTCTGAG	AGCGCGCCATAGTCGTAAGGC
p67phox	CTGTGGTGTGAGCATACGGT	TTAGGCTGAGGCTCCGTAGT
gp91phox	GGGAACTGGGCTGTGAATGA	CAGTGCTGACCCAAGGAGTT
p22phox	AGTGAGGACTTGCGAAGTGG	GAGTAGGCGCCGAAATACCA
p47phox	CCAGGGCACTCTCACTGAATA	ATCAGGCCGCACTTTGAAGAA

Supplementary Table S2. The effects of acute alcohol exposure on mRNA level of housekeeping genes in mouse liver

Housekeeping genes	Cycle numbers		*P* value
	Control	Alcohol	
β-actin	24.39±0.59	22.08±0.52	0.001
Gapdh	17.45±0.82	15.92±0.41	0.016
18S	10.41±0.17	10.49±0.25	0.589

All mice except controls were administered with ethanol (4 g/kg) by gavage. Liver tissue was collected at 6 h after alcohol. The expression of hepatic housekeeping genes were determined using real-time RT-PCR. Data were expressed as means ± SEM (N=6).
